# Effects of 12 Weeks High-Intensity & Reduced-Volume Training in Elite Athletes

**DOI:** 10.1371/journal.pone.0095025

**Published:** 2014-04-15

**Authors:** Anders Kilen, Tanja Hultengren Larsson, Majke Jørgensen, Lars Johansen, Susanne Jørgensen, Nikolai B. Nordsborg

**Affiliations:** 1 Department of Nutrition, Exercise and Sport, University of Copenhagen, Copenhagen, Denmark; 2 Team Denmark, Copenhagen, Denmark; Purdue University, United States of America

## Abstract

It was investigated if high-intensity interval training (HIT) at the expense of total training volume improves performance, maximal oxygen uptake and swimming economy. 41 elite swimmers were randomly allocated to a control (CON) or HIT group. For 12 weeks both groups trained ∼12 h per week. HIT comprised ∼5 h vs. 1 h and total distance was ∼17 km vs. 35 km per week for HIT and CON, respectively. HIT was performed as 6-10×10-30 s maximal effort interspersed by 2–4 minutes of rest. Performance of 100 m all-out freestyle and 200 m freestyle was similar before and after the intervention in both HIT (60.4±4.0 vs. 60.3±4.0 s; n = 13 and 133.2±6.4 vs. 132.6±7.7 s; n = 14) and CON (60.2±3.7 vs. 60.6±3.8 s; n = 15 and 133.5±7.0 vs. 133.3±7.6 s; n = 15). Maximal oxygen uptake during swimming was similar before and after the intervention in both the HIT (4.0±0.9 vs. 3.8±1.0 l O_2_×min^−1^; n = 14) and CON (3.8±0.7 vs. 3.8±0.7 l O_2_×min^−1^; n = 11) group. Oxygen uptake determined at fixed submaximal speed was not significantly affected in either group by the intervention. Body fat % tended to increase (P = 0.09) in the HIT group (15.4±1.6% vs. 16.3±1.6%; P = 0.09; n = 16) and increased (P<0.05) in the CON group (13.9±1.5% vs. 14.9±1.5%; n = 17). A distance reduction of 50% and a more than doubled HIT amount for 12 weeks did neither improve nor compromise performance or physiological capacity in elite swimmers.

## Introduction

High-intensity interval training (HIT), such as 4-6×30 s all-out exercise bouts interspersed by 3–5 minutes of rest, has proved to be a potent stimulus for muscular and cardiovascular adaptation in untrained persons [1] and athletes [2]. In untrained participants, as little as three sessions of HIT per week for 6 weeks causes a ∼7% increase of maximal oxygen uptake (VO_2 max_) and reduces the respiratory exchange ratio ∼0.01 at 65% of VO_2 max_ [3]. Thus, adaptations after HIT are comparable to adaptations after 40–60 minutes of cycling at 65% of VO_2max_ [3]. In highly trained cyclists, VO_2max_ has been found to improve ∼6–8% after HIT [4] but also to be unaffected in other athletic populations such as recreational runners [5]. In addition, HIT can improve work efficiency ∼3–6% in recreational runners and soccer players [5]–[7] which is of importance for performance [8]. With regards to performance, brief intense exercise capacity can be improved ∼7% and time to exhaustion in a prolonged endurance exercise test can be more than doubled in untrained individuals following HIT [3], [9]. In trained individuals performance of brief intense exercise, intermittent running and endurance exercise, such as a 40 km cycling time trial improves ∼5–6% after HIT [2], [4].

At the muscular level in untrained subjects HIT induces mitochondrial biogenesis [9], reduces lactate production [10] and increases capacity for lipid oxidation [3]. In trained subjects, skeletal muscle oxidative enzymatic potential is not always improved [5], [6] but has been observed to increase after one week of HIT in elite distance runners [11]. Thus, the mechanisms responsible for performance improvements with HIT may be different in untrained and trained subjects. There is evidence that HIT leads to a reduction in plasma K^+^ concentration and increased ability to work at high intensities. Reduced plasma K^+^ appears to result from increased skeletal muscle Na^+^, K^+^ pump [5]. However, the role of K^+^ in muscle fatigue is unclear [12]. If HIT improves performance either by inducing mitochondrial biogenesis or by increasing Na^+^, K^+^ pump expression or both, an associated physiological response can be expected. For example, reduced blood lactate concentration, reduced blood acidity and/or reduced blood K^+^ concentrations during standardized exercise could be expected. However, only a very limited number of studies have investigated changes in metabolic markers in response to HIT in elite athletes [11], [13] and no studies have done so when HIT has been performed for more than four weeks. Thus, it is unclear if an added amount of HIT in elite athletes at the expense of total training volume for a prolonged period causes changes in blood markers of metabolism such as pH, lactate and K^+^.

Most HIT studies have investigated interventions lasting from two weeks [3] to ten weeks [2], [14], [15] and it is unknown if HIT at the expense of training volume continues to induce physiological and performance beneficial adaptations if the intervention period is prolonged. In addition, studied populations are either untrained [3] or elite trained without prior systematic engagement in HIT, such as recreational runners [5], [6], [16] or highly trained cyclists [4], [13]. Thus, it also remains unanswered if a substantial increase in the amount of HIT training in an elite population that regularly perform HIT is of similar benefit as populations not previously engaged in HIT. Finally, most studies have included less than ten athletes. Thus, there is a need for studies to include a larger number of subjects in order to detect possibly small differences in outcome measures.

In the present study, performance and physiological adaptations to HIT was investigated in competitive swimmers. Swimmers were selected because HIT training has been a part of competitive swim training for decades [17], however swim training often focuses on a high distance at medium to low intensity [18]. This appears contradictory to the high anaerobic energy demand that exists during competition, where 13 of the 16 Olympic events have race times less than 130 seconds. Thus, competitive swimmers appear to be a good model to investigate if more HIT training at the expense of low intensity high-volume training induces further physiological adaptations and improves performance. A few studies have addressed the impact of HIT in swimming, but these were either performed on children [19], after a 4 months break [20] or without physiological measurements, except from blood lactate [21].

When training volume is drastically reduced in endurance athletes, total caloric intake is not necessarily adjusted accordingly [22]. Additionally, the low total exercise time spent on HIT does not allow the higher intensity to compensate for the reduced total volume in terms of total energy expenditure. Thus, added HIT at the expense of total training distance for a prolonged period of time may increase body weight and/or body fat percentage. This possibility is of obvious importance for elite athletes but has never been addressed in an elite population subjected to HIT at the expense of total training volume. In swimming, added body weight may both be detrimental to performance due to increased frontal area but could also be of benefit due to increased buoyancy.

The purpose of the present study was to investigate if an increased amount of HIT at the expense of a reduced training volume would 1) improve swimming performance; 2) improve swimming specific VO_2max_; 3) improve swimming economy; 4) alter the metabolic response to swimming at a fixed speed and 5) induce increased body fat.

It was hypothesized that the intervention would improve performance, which could be explained by improved swimming economy and increased swimming maximal oxygen uptake and possibly be associated with reduced disturbances of lactate, pH and K^+^ levels at submaximal swimming speeds. Further, it was hypothesized that the reduced training volume would result in elevated body weight due to increased body fat.

## Methods

### Participants

Forty-one healthy Danish national level senior elite swimmers (30 males and 11 females) were recruited for the study. Age: 20.0±2.7 years, height 179.9±6.5 cm and body mass 72.0±10.6 kg. The athletes had been training and competing on a regular basis for a minimum of 5 years, and they were swimming 8–16 hours per week with an average weekly distance of 20.000 m–60.000 m. The enrolled swimmers primarily competed in 50 m–200 m events. Two swimmers were specialized in 400 m and 800 m events. All participants were fully informed of possible risks and discomforts associated with the experimental procedures before they gave their written informed consent to participate. Written informed consent was obtained from one parent if participants were under 18 years. This study conformed to the code of Ethics of the World Medical Association (Declaration of Helsinki) and all procedures were approved by the Ethics Committee of Copenhagen and Frederiksberg communities.

### Intervention Period and Training

An intervention period lasting 12 weeks was carried out in the competitive mid-season from February to May. A two-group parallel longitudinal study design was used. Subjects from four different teams were randomly assigned to either an intervention group (HIT group; n = 20, 14 males and 6 females) or control group (CON group; n = 21, 16 males and 5 females). From each team, swimmers were assigned to both HIT and CON groups. In the HIT group, regular training volume was reduced by 50% and the amount of high intensity training was more than doubled. In table 1, a simplified summary of training data is provided based on team coaches' registration and training reports from participating swimmers. In the CON group, training was continued as usual. All training sessions were supervised and logged by team coaches who had received detailed instructions by the research team. Additional dry-land training with focus on core-stability was performed for approximately 20 minutes per day and strength training with focus on upper body strength was performed for up to 2 hours per week. Participating swimmers were instructed to maintain their regular eating habits but this was not controlled.

**Table 1:** Training intensity and volume.

	Total volume and hours		Li -Aerobic		Hi - Aerobic		HIT	
Week	CON	HIT	CON	HIT	CON	HIT	CON	HIT
PRE	13 h	13 h	10.7 h	10.7 h	1.3 h	1.3 h	1.0 h	1.0 h
	34 km	34 km	31.7 km	31.7 km	1.5 km	1.5 km	0.8 km	0.8 km
1–2	13 h	13 h	10.7 h	6.7 h	1.3 h	1.3 h	1.0 h	5.0 h
	34 km	17 km	31.7 km	12.0 km	1.5 km	1.0 km	0.8 km	4.0 km
3–4	20 h	20 h	16.4 h	10.4 h	2.0 h	2.0 h	1.6 h	7.6 h
	52 km	26 km	48.4 km	17.6 km	2.3 km	2.3 km	1.3 km	6.1 km
5–6	13 h	13 h	10.7 h	6.7 h	1.3 h	1.3 h	1.0 h	5.0 h
	34 km	17 km	31.7 km	12.0 km	1.5 km	1.0 km	0.8 km	4.0 km
7–8	13 h	13 h	10.7 h	6.7 h	1.3 h	1.3 h	1.0 h	5.0 h
	34 km	17 km	31.7 km	12.0 km	1.5 km	1.0 km	0.8 km	4.0 km
9–10	11 h	11 h	8.5 h	5.0 h	1.0 h	1.0 h	1.5 h	5.0 h
	24 km	12 km	22.0 km	7.2 km	0.8 km	0.8 km	1.2 km	4.0 km
11–12	13 h	13 h	10.7 h	6.7 h	1.3 h	1.3 h	1.0 h	5.0 h
	34 km	17 km	31.7 km	12.0 km	1.5 km	1.0 km	0.8 km	4.0 km

Training time and milage averaged for every 2^nd^ week and split into three major training categories. CON: Control groups; INT: intervention group. Li-Aerobic: Technical training, Recovery and low to moderate aerobic training with % of maximal heart rate < 70%. Hi-Aerobic: Intense aerobic training aiming at eliciting close to maximal heart rate and >90% of VO_2max_. HIT: “High-Intensity Training” with maximal effort for 20–90 s and a rest: work ratio > 4.

### Experimental protocols

Before (PRE) and after (POST) the HIT intervention period, participants underwent a series of physiological evaluations: body composition analyses; determination of swimming economy and swimming peak oxygen uptake in a custom built swim flume; a pool based 5×200 m freestyle swim test with increasing speeds and blood analyses. Additionally, performance was evaluated by analyses of 100 m freestyle all-out and 200 m freestyle completed in competition. Details for each procedure are given below. All subjects completed familiarization to all physiological tests six weeks before PRE-testing. The familiarization trial; PRE-trial and POST-trials followed the same time schedule so that an individual underwent testing at the same time of day on all occasions. First experimental day was always a Friday and the second experimental day was either the following Saturday or Sunday. Swimmers were instructed not to do any training on the experimental days prior to testing. Swimmers were also instructed to eat at least two hours before the first test and to keep a record of food intake. Participants received written and verbal instructions to repeat the food consumption schedule on the three test occasions. For logistic reasons, several of the enrolled swimmers did not complete the evaluations. Thus, the number of evaluated swimmers is given for each reported variable.

### Laboratory analyses

On the first experimental day, body composition was determined by Dual x-ray absorptiometry (DEXA) scanning (Lunar Prodigy, GE Healthcare, UK). Subsequently, swimming economy and maximal oxygen uptake during freestyle swimming was measured in a custom built swimming flume. Pulmonary oxygen uptake and ventilation were measured breath-by-breath (Cosmed Quark b2, Milan, Italy). Swimmers completed a five minutes warm-up at submaximal speed chosen so that the swimmers reported the swim to be “easy”. Subsequently, swimming economy was analyzed by completion of a six minutes continuous swim at the same speed as during warm-up. Based on the pre-test results, the speed was selected to represent ∼60% of VO_2 max_. After three minutes of rest, a progressive test to exhaustion was completed with speed increasing ∼0.1 m×s^−1^ every minute. The duration of the incremental test varied between four and eight minutes. The incremental test design was based on pilot studies, where it was found that longer test durations caused premature fatigue.

### Pool testing

On the second experimental day, pool tests were completed in a 25 m six lane pool. All testing was performed using freestyle technique. First, the swimmer completed a warm-up of ten minutes followed by 5×200 m at increasing pace. The pace was determined so that the first three 200 m were submaximal (subjectively rated as “very easy” to “somewhat hard”). For the first three 200 m, two groups were designed based on individual level in the initial testing. One group paced at 160.2±1.7 s; 155.5±1.9 s and 150.6±1.9 s. The other group paced at 170.0±1.0 s; 165.3±1.9 s and 160.8±1.0 s. The final two 200 m were paced individually (subjective rating “hard, but not maximal” and “maximal”) with average pacing of all trials being 138.0±5.6 s and 130.0±5.9 s in the first group and 149.2±5 s and 142.6±7.3 s in the second group. The same pace scheme was used on all test occasions. Pacing was controlled by the scientific staff with audio signals given every 25 m. Each 200 m was started in the water from the wall and swimmers used tumble turns. One minute after each swim, a finger capillary blood sample was obtained and used for analyses of blood lactate concentration (YSI 1500 Sport, Yellow Springs Instruments, USA). Additionally, after the first, third and fifth 200 m and three minutes after the fifth 200 m an additional capillary sample was obtained and analyzed for blood pH and [K^+^] (GEM premier 4000). Arm stroke frequency and swim time was registered for each 200 m using a stop watch with frequency count function (Seiko S141, Tokyo, Japan).

After 4½ hours of recovery, swimmers did a 15-minute warm-up followed by an all-out 100 m freestyle. Subjects started with a standardized push off from the wall. To avoid pacing and racing strategies, the 100 m was completed by one swimmer at a time. After 20 minutes of recovery (range from 15–25 minute), the swimmers completed a 200 m freestyle at competition like conditions with heats of three swimmers and start from the block. In this analysis, subjects were matched according to expected best time.

### Statistics and calculations

Statistical analyses were performed using SPSS, Statistics 20.0. Data distribution and variance was visually inspected and found to be normally distributed with homogeneous variance between groups. Differences between groups were analyzed using a mixed model [23] with fixed factors: ”Group” (Control, Intervention); “Trial” (PRE, POST) and subject specified to identify repeated observations. If significant main effects or interactions were present post hoc test were applied by use of t-tests with Bonferroni adjustment. The level of significance was set to p<0.05. Results are reported as means ± standard deviations (SD). The number of participants varies between analyses due to either logistic or technical difficulties. The number of participants is reported for each analysis. An inference about population effects approach was applied to the primary outcome variables of the present study [24]. The change from pre to post was calculated and evaluated relative to 0 by a one-sample t-test. A 90% confidence interval was calculated and the probability of a beneficial or harmful effect was inferred by defining threshold values and using a t-distribution.

## Results

### Performance

**Figure 1:**
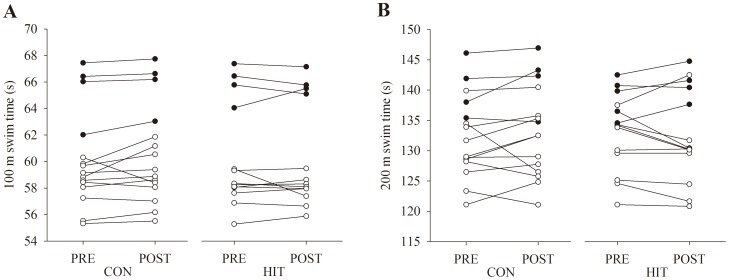
Swimming performance. Performance of 100-out, starting with wall push-off and one swimmer in the pool at a time (A) and 200 m freestyle in a competition setting, starting from blocks in heats of 3–4 swimmers (B) as determined before (PRE) and after (POST) a training intervention. One group performed usual swim-training (CON) and another group reduced their training volume by 50% and more than doubled the amount of high-intensity training (HIT). On average, both the 100 m and 200 m performance was similar before and after the 12 week intervention period in both groups. Individual data is presented. Open circles: male; Closed circles: female.

Performance of 100 m all-out freestyle (Fig. 1A) was similar before and after the intervention (Trial: p = 0.34; Group: p = 0.97; Trial×Group: p = 0.16) in both the HIT (60.4±4.0 s vs. 60.3±4.0 s; n  =  13; p = 0.75) and CON (60.2±3.7 s vs. 60.6±3.8 s; n = 15; p = 0.09) group. Likewise, performance of 200 m freestyle (Fig. 1B) in simulated competition was similar (Trial: p = 0.65; Group: p = 0.97; Trial×Group: p = 0.99) before and after the intervention in both the HIT (133.2±6.4 s vs. 132.6±7.7 s; n = 14; p = 0.75) and CON (133.5±7.0 s vs 133.3±7.6 s; n = 15; p = 0.75) group. Also, average speed of a 200 m freestyle performed after four preceding 200 m swims with increasing speed was similar (Trial: p = 0.36; Group: p = 0.32; Trial×Group: p = 0.48) before and after the intervention in both the HIT and CON group (1.48±0.10 m×s^−1^ vs. 1.50±0.08 m×s^−1^; n = 15; p = 0.26 and 1.52±0.09 m×s^−1^ vs. 1.52±0.09 m×s^−1^; n = 16;p = 0.88). Stroke- rate and length was similar (Trial: p = 0.39; Group: p = 0.52; Trial×Group: p = 0.65) during the paced 200 m before and after the invention in both the HIT (29.9±2.3 strokes×min^−1^ vs. 29.8±2.3 strokes x min^−1^; n = 15; p = 0.70) and CON (29.4±3.3 strokes×min^−1^ vs. 29.0±3.6 strokes×min^−1^; n = 16; p = 0.43) group.

### Maximal oxygen consumption during flume swimming

**Figure 2:**
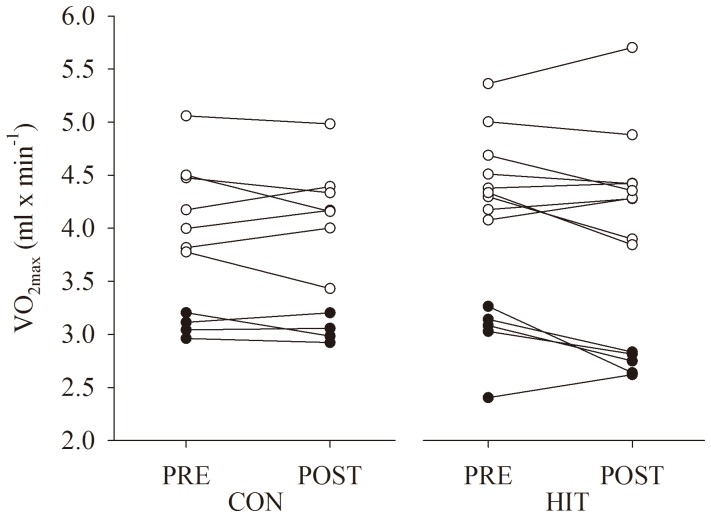
Oxygen uptake. Maximal oxygen uptake (VO_2max_) determined during freestyle swimming in a swimming flume with increasing water speed until exhaustion in two groups of swimmers before (PRE) and after (POST) a training intervention. One group performed usual swim-training (CON) and another group reduced their training volume by 50% and more than doubled the amount of high-intensity training (HIT). On average, VO_2max_ was similar before and after the 12 week intervention period in both groups. Individual data is presented. Open circles: male; Closed circles: female.

VO_2 max_ determined during freestyle swimming with increasing speed in a flume (Fig. 2) was similar (Trial: p = 0.08; Group: p = 0.76; Trial×Group: p = 0.35) before and after the intervention in both the HIT (4.0±0.9 lO_2_×min^−1^ vs. 3.8±1.0 lO_2_×min^−1^; n = 14; p = 0.09) and CON group (3.8±0.7 lO_2_×min^−1^ vs. 3.8±0.7 lO_2_×min^−1^; n = 11; p = 0.56). In contrast, VO_2 max_ expressed relative to body weight was affected by the intervention (Trial: p = 0.01; Group: p = 0.95; Trial×Group: p = 0.26) with a decrease in HIT (55.7±7.2 mlO_2_×min^−1^×kg^−1^ vs. 52.7±7.0 mlO_2_×min^−1^×kg^−1^; n = 14; p = 0.02) and no significant difference in CON (55.0±5.9 mlO_2_×min^−1^×kg^−1^ vs. 53.8±6.4 mlO_2_×min^−1^×kg^−1^; n = 13; p = 0.31).

### Swimming economy

VO_2_ determined at a fixed submaximal speed before and after the intervention was similar (Trial: p = 0.37; Group: p = 0.37; Trial×Group: p = 0.74) in both the HIT (2.4±0.7 lO_2_×min^−1^ vs. 2.4±0.7 lO_2_×min^−1^; n = 13; p = 0.39) and CON (2.6±0.6 lO_2_×min^−1^ vs. 2.6±0.6 lO_2_×min^−1^ n = 14; p = 0.68) groups.

### Ventilation

V_E_ determined at a fixed submaximal speed before and after the intervention was similar (Trial: p = 0.17; Group: p = 0.58; Trial×Group: p = 0.92) in both the HIT (63.0±26.9 l×min^−1^ vs. 61.4±27.1 l×min^−1^; n = 15; p = 0.40) and CON (58.9±13.5 l×min^−1^ vs. 57.0±11.1 l×min^−1^; n = 15; p = 0.26) groups. V_E_×VO_2_^−1^ determined at a fixed submaximal speed before and after the intervention was similar (Trial: p = 0.59; Group: p = 0.29; Trial×Group: p = 0.95) in both the HIT (24±4 l×min^−1^ vs. 23±4 l×min^−1^; n = 15; p = 0.67) and CON (24±4 l×min^−1^ vs. 22±4 l×min^−1^; n = 15; p = 0.73) groups.

### Respiratory exchange ratio

The respiratory exchange ratio determined at a fixed submaximal speeds before and after the intervention was similar (Trial: p = 0.47; Group: p = 0.37; Trial×Group: p = 0.61) in both the HIT (0.87±0.04 vs. 0.87±0.04; n = 13; p = 0.88) and CON (0.88±0.02 vs. 0.88±0.04; n = 13; p = 0.38) groups.

### Blood lactate, pH and K^+^ response to increasing swimming speed

**Figure 3:**
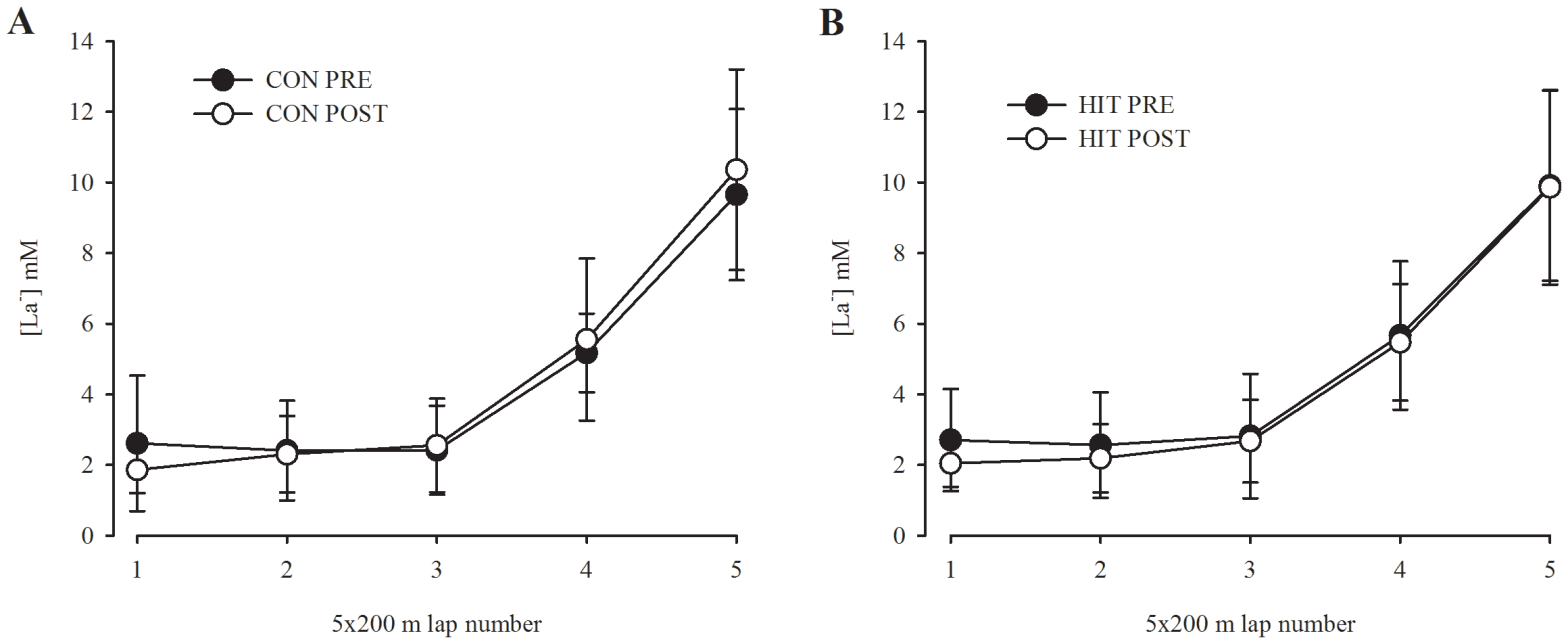
Blood lactate. Blood lactate determined from a finger capillary sample obtained 1 minute after each of five 200(see methods) in two groups of swimmers before (PRE) and after (POST) a training intervention. One group performed usual swim-training (CON) and another group reduced their training volume by 50% and more than doubled the amount of high-intensity training (HIT).

Blood lactate response to increasing swimming speeds during five times 200 m freestyle was similar (Trial: p = 0.60; Group: p = 0.90; Sample: p<0.001; Trial×Group×Sample: p = 0.31) before and after the intervention in both the HIT and CON group (Fig. 3). Furthermore, the change in blood lactate concentration from the 3^rd^ 200 m to the sample obtained after the 5^th^ 200 m (Table 2) was similar (Trial: p = 0.66; Group: p = 0.32; Trial×Group: p = 0.55). Blood pH response to the third and fifth swim interval was similar (Trial: p = 0.81; Group: p = 0.74; Trial×Group: p = 0.82 and Trial: p = 0.62; Group: p = 0.25; Trial×Group: p = 0.77, respectively) before and after the intervention in both groups (Table 2). Likewise, blood K^+^ response to the third and fifth swim interval was similar (Trial: p = 0.89; Group: p = 0.57; Trial×Group: p = 0.74 and Trial: p = 0.36; Group: p = 0.74; Trial×Group: p = 0.99, respectively) before and after the intervention in both groups (Table 2).

**Table 2:** Blood metabolites.

		Intervention group			Control group		
Blood							
variable	Test	Pre	Post	n	Pre	Post	n
pH	3^rd^ 200 m	7.39±0.04	7.40±0.04	15	7.39±0.04	7.39±0.03	16
pH	5^th^ 200 m	7.23±0.06	7.22±0.07	15	7.20±0.09	7.20±0.07	17
ΔpH	3^rd^ → 5^th^	0.17±0.05	0.18±0.06	15	0.19±0.07	0.19±0.06	16
ΔLactate	3^rd^ → 5^th^	7.8±2.7 mM	7.2±2.6 mM	15	8.2±2.6 mM	8.3±2.6 mM	17
K^+^	3^rd^ 200 m	4.78±0.35 mM	4.74±0.44 mM	15	4.69±0.23 mM	4.66±0.36 mM	16
K^+^	5^th^ 200 m	5.15±0.53 mM	5.01±0.61 mM	15	5.09±0.88 mM	4.95±0.56 mM	17
ΔK^+^	3^rd^ → 5^th^	0.43±0.54 mM	0.27±0.67 mM	15	0.38±0.85 mM	0.34±0.56 mM	16

Blood variables obtained from a fingertip sample after the 3^rd^ and 5^th^ of five 200 m swims performed at increasing speeds before and after a 12 week intervention period. The third 200 meter was swum on a identical speed before and after the intervention whereas the fifth 200 meter was swum with maximal effort.

### Body composition

Body fat percent as determined by DEXA scanning was affected by the intervention (Trial: p = 0.01; Group: p = 0.51; Trial×Group: p = 0.99). For the HIT group (n = 16) the increase did not reach statistical significance (15.4±1.6% vs. 16.3±1.6%; p = 0.09). In the CON group (n = 17) body fat percent increased from 13.9±1.5% to 14.9±1.5% (p = 0.04).

Results from the inference about population effects approach is presented in Table 3.

**Table 3:** Inference of effects.

	100 m	200 m	VO_2 max_	VO_2 sub_	RER _sub_	[La^-^]_max_	pH _max_	[K^+^]_max_
HIT								
90% C.I.	[−0.3;0.5]	[−0.9;2.0]	[−280;−5]	[−110;33]	[−0.02;0.02]	[−0.66;1.3]	[−0.03;0.01]	[−0.5;0.22]
P	0.72	0.48	0.09	0.44	0.90	0.59	0.49	0.51
Threshold	0.2 s	0.2 s	150 ml	−100 ml	−0.02	0.5 mM	0.02	0.2 mM
Inference								
- Beneficial	31%	68%	0%	9%	5%	36%	22%	6%
- Trivial	57%	15%	53%	91%	88%	56%	76%	55%
- Harmful	12%	17%	47%	0%	7%	8%	2%	39%
CON								
C.I.	[−0.9;0.0]	[−2.3;0.7]	[−150;67]	[−110; 68]	[−0.02;0.01]	[−0.39;1.9]	[−0.04;0.03]	[−0.52;0.24]
P	0.12	0.375	0.49	0.707	0.285	0.267	0.901	0.525
Threshold	0.2 s	0.2 s	150 ml	−100 ml	−0.02	0.5	0.02	0.2 mM
Inference								
- Beneficial	1%	13%	0%	6%	9%	65%	18%	7%
- Trivial	19%	12%	94%	92%	91%	32%	70%	54%
- Harmful	80%	75%	6%	2%	0%	3%	12%	39%

The 90% confidence interval (90% C.I.) for the observed change in primary outcome variables within each group and the P-value (P) for a one-sample t-test with the null-hypothesis µ = 0. A subjectively chosen threshold for the minimal change that could be considered beneficial (Threshold) was used to calculate the chance that the observed changes would be beneficial, trivial or harmful (Inference). One group performed usual swim-training (CON) and another group reduced their training volume by 50% and more than doubled the amount of high-intensity training (HIT).

## Discussion

The major findings were that more than a doubling of high-intensity training (HIT) in combination with a 50% reduction of training volume for 12 weeks did not change swimming performance, swimming specific VO_2max_, swimming economy, blood metabolic markers or body composition as compared to a control group. However, VO_2max_ normalized to body weight was reduced in the HIT group only.

The current observation that added HIT and reduced training volume for 12 weeks does not improve performance as compared to high volume training is in agreement with a previous study of ten elite swimmers, where 6.5 weeks of HIT after a four months break yielded similar performance improvements as in a control group [20]. The result is also consistent with unaltered performance after 5 weeks in-season HIT training in younger (∼16-17 years) swimmers [21]. However, in children (∼10 years) 5 weeks of HIT improved swimming performance in competition [19] indicating that the investigated age group may be of importance. The unaltered performance after HIT in adult swimmers is in contrast to recreational runners who improved time to exhaustion in tests lasting 1–3 minutes [5], [16], [25] and ∼5–6 minutes [15]. Furthermore, in athletes that have not previously engaged in HIT performance can also be improved by added HIT and reduced volume [26], [27]. Because the swimmers investigated in the present study had performed HIT for a number of years as a part of their regular training, an upper limit may exist to the amount of HIT that can be applied and still yield physiological adaptation and performance enhancement. In this context it may be of importance to consider that technique in swimming is a major determinant of performance [28] and thus HIT induced physiological adaptations may be less important in swimming as compared to for example running or cycling. Notably, the reduction of training volume in the CON group did not compromise performance. Thus, the increased amount of HIT allowed a drastic distance reduction without compromising performance. The reduced distance and increased recovery time is important for yearly training planning because periodic HIT may allow time to other important training focuses such as technical and/or tactical skills.

The present observation of unaltered swimming specific VO_2max_ after a prolonged period of HIT training is in accordance with observations from moderately trained runners [5], [6] but in contrast to the increase observed in well-trained cyclists after 4 weeks of added HIT [13] and in moderately trained cyclist after 6 weeks of HIT [29] as well as findings from untrained subjects [3]. There are several possible explanations for the observed unaltered VO_2max_. Importantly, the current population had a much higher training volume and longer history than previously studied populations. Thus, a natural plateau in VO_2max_ may have been reached prior to the initiation of the study. This suggestion is somewhat supported by the observation that HIT induces similar increases in VO_2max_ as low-intensity high-volume training in young swimmers aged ∼10 years [19] as well as in adult swimmers when preceded by a four months break [20]. It may also be considered that swimming VO_2max_ may not have been limited by the central cardiovascular capacity as expected during running or cycling [30] but more so by the upper body capacity to extract oxygen. This suggestion is substantiated by the finding that seasonal fluctuations in a Olympic level swimmers VO_2peak_ appears unrelated to running VO_2peak_ [31]. As such, the current finding of unaltered VO_2max_ may indicate unaltered muscular respiratory capacity and this suggestion is in agreement with a previous study that found unaltered muscular oxidative capacity in trained populations after a period of HIT [2]. It is of interest to note that the previously reported improvement of running economy, which was associated with reduced VO_2_ but unchanged RER at submaximal speeds [6] could not be verified in the present study of elite athletes. Importantly, a reduced VO_2max_ relative to body weight was apparent in the HIT group but not the CON group. However, it could not be demonstrated that the change in normalized VO_2max_ differed between groups. Further, both groups experienced a gain of body weight of ∼1 kg which was not significantly different between groups (data not shown).

The present investigation of blood metabolic markers in response to increasing swimming speed demonstrated the expected increases of blood lactate, K^+^ and H^+^ concentrations. However, the hypothesis of reduced extracellular accumulation of blood lactate, K^+^ and H^+^ in the HIT group after the intervention period could not be verified. This is in apparent contrast to the observation that HIT can increase muscle Na^+^, K^+^ ATPase expression and reduce plasma K^+^ concentration at the end of 1–2 minutes exhaustive runs in recreational runners [5], [32]. After HIT plasma K^+^ may also be reduced during the recovery from exercise [32], [33], but this is not always the case [5]. In the present study, samples were obtained one minutes after an exhaustive 200 m swim that lasted ∼2 minutes, which corresponds well to the samples obtained during early recovery in previous studies [16], [33]. The present observations are in accordance with unaltered plasma K^+^ concentrations after HIT during submaximal workloads and after an incremental test [5]. It can be speculated that reduced plasma K^+^ concentrations may have existed in the present study if the swimmers had been evaluated after a constant speed swim to exhaustion [5]. However, this type of test does not correspond to the competition performed by swimmers and most other athletes.

During the 5×200 m pool test with gradually increasing swimming speed, blood lactate concentration was unaltered. Additionally, the change in blood lactate concentration from the 3^rd^ to the 5^th^ 200 m was unaltered by the intervention, indicating that the muscular lactate production and release was similar before and after HIT. This is supported by the unaltered changes in plasma pH observed during the 5×200 m steptest before and after the HIT period. The current observations are in agreement with the finding that blood and muscle lactate as well as muscle H^+^ accumulation and muscular expression of monocarboxylate transporters (MCT) 1 and 4 is unaltered after HIT in recreational runners [5], [16]. However, muscular MCT1 expression has also been observed to increase after HIT in recreational runners [25] and the reason for this discrepancy between previous studies is unclear but may be related to differences in the applied HIT protocols.

Metabolites were measured in plasma during recovery in the present study and it cannot be excluded that undetected changes in other compartments and at other time-points existed. Previous studies have demonstrated that HIT reduces K^+^ accumulation in the interstitial space in untrained subjects [34] and that changes in K^+^ plasma concentrations occurs within 10–30 s after onset and termination of exercise [35], [36] and that plasma K^+^ concentration is sensitive to training [37]. Additionally, muscle lactate and H^+^ accumulation can be reduced by HIT [38] and with respect to sample timing, exercise induced changes in lactate and H^+^ homeostasis persist for several minutes into recovery in both intramuscular [39] and in the plasma compartment [38]. Thus, training induced changes in muscular capacity for handling K^+^, H^+^ and lactate usually translates into altered plasma accumulation [34] and is detectable during recovery, which suggests that the present observations of unaltered plasma metabolite response is representative for the events at the muscular level.

Only the CON group showed an increase of body fat during the intervention period, but a similar and close to significant increase was apparent in the HIT group. This finding demonstrates that a drastic shift in training volume does not necessarily translate into to altered body composition. This observation is in agreement with unaltered body composition, despite a one week change in total daily energy expenditure in trained athletes [22].

The present study is the longest running HIT intervention in an elite-population and at the same time includes more subjects than most previous studies [2], [18]. Despite the long intervention period, relatively large group of subjects and carefully controlled training and testing, some concerns need attention. It may be speculated that some, but not all, swimmers can benefit from HIT and if there is any chance that HIT can improve performance of some but not all elite athletes, then it is of interest for coaches and athletes. Thus, we report results of an inference statistical approach [40] for the primary outcome variables in Table 3. This analysis supported that no clear beneficial or harmful effect was apparent after 12 weeks of HIT. It should be noted that performance of 100 m and 200 m freestyle may have been impaired in the CON but not in the HIT group whereas VO_2max_ may have decreased in the HIT group but not in the CON group. These observations may be considered in a practical setting depending on the type of swimmer that is exposed to HIT. Apparently, HIT may be of some value to protect against reduced performance in short distance swimmers but based on the possible reduction in VO_2max_, it can be speculated that HIT would be detrimental for long distance swimmers (i.e. > 800 m) but this remains to be investigated. The possible reduction of VO_2max_ is somewhat supported by the low p-values observed for pre vs. post VO_2max_ and the reduced VO_2max_ normalized to body weight in the HIT group. Another possible concern is that all testing was completed in freestyle for practical reasons. It cannot be ruled out, that for example, breast swimmers benefitted from the training whereas back-strokers did not. However, all swimmers completed more than 50% of the total training volume in freestyle, including the HIT sessions. Thus, this appears a reasonable but negligible concern. It can also be speculated that the CON group achieved optimal adaptations resembling those of HIT by the performed in-water HIT, dry-land resistance training or even less intense but prolonged interval based training that possibly could cause adaptations as fatigue sets in. Even if the CON group somehow did achieve adaptations similar to the HIT group the present findings still demonstrate that a drastic distance reduction for 12 weeks does not compromise performance. Thus, it becomes of interest to investigate if a drastic distance reduction combined with a limited amount of HIT would also be sufficient to sustain performance for a prolonged period of time.

Both genders participated in the present study but the number of subjects was too limited to allow for a separate analysis of possible gender specific adaptation to the intervention. Previously, it has been demonstrated that recreationally active young men and women adapted similar to a 2–3 week period of HIT [41] but it is not known if a gender effect exists in an elite population.

### Perspective

The present data demonstrate that it is possible to reduce training volume by 50% without compromising physical capacity. Because time is limited in elite training HIT can be used as a strategy to increase the focus on other types of training because of the long recovery times and reduced time requirement. The present findings indicate that for example technical and tactical focus can be increased for periods of up to 12 weeks during the season by reducing volume and increasing the amount of HIT. In a physiological context, the present data demonstrate that added HIT to a very well trained population all-ready engaging in HIT does not necessarily improve performance as could be expected from previous studies of HIT.

### Conclusion

In conclusion, increasing the amount of HIT at the expense of volume for 12 weeks in a group of elite swimmers did neither induce significant improvements nor deterioration of performance or physiological variables.
